# Falls prevention through physical and cognitive training (falls PACT) in older adults with mild cognitive impairment: a randomized controlled trial protocol

**DOI:** 10.1186/s12877-018-0868-2

**Published:** 2018-08-24

**Authors:** Donald S. Lipardo, William W. N. Tsang

**Affiliations:** 1 Department of Rehabilitation Sciences, The Hong Kong Polytechnic University, Hung Hom, Kowloon, Hong Kong, SAR China; 2 Department of Physical Therapy, College of Rehabilitation Sciences, University of Santo Tomas, Manila, Philippines

**Keywords:** Accidental falls, Older adults, Mild cognitive impairment, Physical exercise, Cognitive training, Falls rate

## Abstract

**Background:**

The presence of mild cognitive impairment (MCI) in older adults increases their fall risk. While physical exercise is effective in reducing falls rate and risk of falls, and cognitive training in improving cognitive functioning in healthy older adults, their effectiveness in preventing falls and reducing risks of falls in MCI when administered simultaneously is not yet established. Therefore, this study aims to determine the effectiveness of combined physical and cognitive training in preventing falls and decreasing risks of falls among community-dwelling older persons with MCI.

**Methods/design:**

This is a single-blind, multicentre, randomized controlled trial. At least ninety-three community-dwelling older adults with MCI aged 60 or above will be recruited. They will be randomly allocated into four groups: Physical Training alone (PT), Cognitive Training alone (CT), combined Physical And Cognitive Training (PACT) and Waitlist Group (WG). The PT group will perform exercises (flexibility, endurance, strengthening, and balance training) for 60–90 min three times per week for 12 weeks. The CT group will be involved in a paper-based training focusing on orientation, memory, attention and executive functioning for 60–90 min per session, once a week for 12 weeks. The PACT group will undergo cognitive training incorporated in physical exercise for 60–90 min three times per week for 12 weeks. The WG will receive the intervention, combined physical and cognitive training, at a later date. Assessors blinded to participant allocation will conduct pre-intervention, post-intervention, and 6-month follow-up assessments. The primary outcome measure will be falls rate. The secondary outcome measures will be Physiologic Profile Assessment and Falls Risk for Older Persons in the Community, and assessments that evaluate cognitive, physical and psychological factors related to falls.

**Discussion:**

Considering the possible physical, social, financial and psychological consequences of a fall, we hope to provide insights on the effectiveness of combining physical and cognitive training on falls and fall-related factors for older adults with MCI. It is projected that the combined interventions will lead to significantly lower falls rate and reduced risk of falls compared to using single or no intervention.

**Trial registration:**

ClinicalTrials.gov NCT03167840. Registered on May 30, 2017.

## Background

Mild cognitive impairment (MCI) is associated with falls in the geriatric population [1], particularly among female older adults [2]. It is the intermediary state of cognitive decline between the changes due to the normal aging process, and the deterioration due to dementia and other neurological diseases [3]. The estimated global prevalence rate of MCI is between 5.0 and 36.7% [4].

MCI is considered a predictor of falls [5] but it is also a potentially ameliorable fall risk factor [6]. Interventions, including physical exercise and cognitive training [7], may be provided to address the decline in cognitive function and also the presence of some physical fall-related risk factors such as decreased balance control [8], muscle weakness [9] and slow gait [10] that predisposes older adults to incur a fall.

### Physical training

Physical exercise is one of the most common interventions used to prevent falls in the geriatric population [11, 12]. Exercise as a single intervention or as a component of a multifactorial intervention has been proven effective in reducing rate of falls and risk of falls among the general population of older adults living in the community [11, 12]. To successfully reduce falls incidence in older people, a minimum of three hours every week [11] for at least 40 h of exercise over the course of interventions is needed [13]. The optimal exercise frequency is purported to be three times per week [13]. Designing the program to comprehensively include balance, strength, endurance, and flexibility, but with at least one-third is focused on balance training, is recommended [11–13].

For community-dwelling older adults with cognitive impairment, there is promising evidence that exercise as a standalone intervention may prevent the occurrence of falls, however, this entails confirmation with more trials [11]. Older adults with MCI, in particular, are faced with certain cognitive hindrances when engaging in exercise such as learning new routines and remembering how to perform them accurately [14]. These cognitive obstacles were observed in spite of previous involvement in similar exercise regimens [15].

In terms of the effect of exercise on cognitive function, contrasting findings were reported. One meta-analysis concluded that there is very limited evidence that exercise improves cognitive function in MCI [16]. A more recent systematic review, however, reported positive effects of exercise on global cognition, executive function, attention and delayed recall in older persons with MCI [17]. Specifically engaging in aerobic exercise (walking, dancing, jogging, Tai Chi) at 60–80% maximum heart rate or > 3 metabolic equivalents has resulted to improved cognitive ability of older adults with MCI [18]. On the other hand, doing functional tasks as a form of exercise has been found to provide cost-effective and sustainable improvements in global cognitive function, memory and executive function in community-dwelling older persons with MCI [19]. These benefits were obtained with the use of physical exercise alone and without formal cognitive training of the participants.

### Cognitive training

Cognitive training, which usually involves guided practice on a set of specific tasks designed to solicit targeted cognitive functions [20], has been proven effective in healthy older adults in improving cognitive functioning [21–23] and may also be an effective preventive strategy in the onset of any cognitive decline among older persons [24]. Impairment in executive function is found to be independently associated with a heightened risk for falls and fall-related injuries in community-dwelling older adults [25, 26]. Executive function (EF) is a high-level subcomponent of cognition that regulates processes including working memory, inhibition and cognitive flexibility which are important in adapting efficiently to changing environment and task [27]. Targeting particularly executive dysfunction in cognitive training has been recommended to decrease the rate of falling in the geriatric population with cognitive impairment due to Alzheimer’s disease [28]. This strategy may help decrease falls rate in Alzheimer’s disease by addressing the perceptual-motor integration problem that causes instability in performing certain automatic tasks such as walking that eventually results in a fall [29].

In older adults with MCI, cognitive training has resulted in beneficial effects on cognitive function such as attention, orientation, perception, language, memory and executive function [23, 29]. However, there are a lot of inconsistencies in terms of the format (group versus individual), number and duration of treatment sessions in the previously implemented programs [23, 30]. It was found out that there is no dose-response relationship between the total training hours and the effectiveness of intervention on cognitive outcomes [23]. But, it seems that fewer sessions, about 6–20 sessions only, are considered more cost-effective [30]. Furthermore, cognitive training greater than 12 weeks duration did not show better outcomes compared to programs with ≤12 weeks duration, where the risk of attrition is also reduced [30].

### Combined physical and cognitive training

A treatment paradigm that could potentially address both cognitive decline and risk of falls in MCI is to combine physical exercise and cognitive training. Neuroplastic changes in the brain may be more evident if these two interventions are integrated. In an animal study, it was suggested that physical activity and cognitive stimulus had a complementary effect on neurogenesis [31]. In humans, combined physical/cognitive training had an effect on brain functional plasticity. The Train the Brain Consortium (2017) reported that there were lesser neural resources utilized, which means better neural efficiency, for the same behavioral activities in older individuals with MCI on combined interventions training compared to those without training [32]. Aside from neural efficiency, increased cerebral blood flow in the parahippocampal area in the medial temporal lobe of the brain was more evident after combined interventions. This area of the brain subserves non-verbal spatial information processing [32].

Among healthy older adults, combined interventions in the form of stationary cycling with virtual reality [33], and integrated aerobic exercise and mental training [34] resulted in better effects in their cognitive performance compared when the interventions were delivered separately. Similarly, progressive balance training with dual and multi-task exercises lead to positive short and long-term benefits in gait, balance control and fear of falling among community-dwelling healthy older adults with increased risk of falling [35].

For older adults with cognitive impairment, the effect of combined physical and cognitive training is rather inconsistent. In older persons with Parkinson’s disease, the multimodal cognitive program, including cognition, transfer training, psychomotor and endurance, had superior effects in cognitive function compared to paper-and-pencil-based cognitive training only [36]. In older adults with MCI, cognitive status was significantly improved following a 7-month combined physical-cognitive training and music therapy compared to no training [32]. Simultaneous physical and cognitive training by dual-tasking in older persons with MCI also resulted in significantly better cognitive outcomes compared to a waitlist group [37] or an education control group [38].

On the contrary, a study that combined resistance training and cognitive training in older adults with MCI concluded that doing high-intensity resistive training alone for six months lead to significant increase in global cognitive function, memory and executive function while participating in combined training unexpectedly and significantly reduced the benefits of isolated progressive resistance training on executive and global cognitive function [39]. In another study also involving older persons with MCI, dual modality cognitive-physical training and single modality interventions of cognitive training alone or physical exercise alone resulted in no significant differences in cognitive outcome measures [40]. In a systematic review, both older adults with and without cognitive impairment gained improvements in cognitive functions and functional status from combined physical exercise and cognitive training interventions [41]. However, the evidence is lacking to ascertain the superiority of combined interventions when compared to active control groups. More studies with good methodological quality are warranted to explore the potential benefits of this new treatment approach [41].

The link connecting the improvements in cognitive function and physical factors after undergoing physical exercise and cognitive training, singly delivered or in combination, to the reduction of falls and the decrease in falls risk in the geriatric population, particularly to those with MCI, is yet to be established.

Thus, this study primarily aims to determine the effectiveness of combined physical and cognitive training on preventing falls and reducing the risk of falls among community-dwelling older adults with MCI. The hypothesis is that an intervention that incorporates elements of both physical exercise and cognitive training in one treatment program is more effective compared to physical exercise or cognitive training alone in preventing the occurrence of falls and in decreasing the risk of falls in older adults with MCI living in the community.

## Methods

### Participants

Community-dwelling older adults, both male and female, aged 60 or above with MCI will be included. They will be recruited from community sites in Manila, Philippines through the assistance of local coordinators of the Office of Senior Citizens Affairs (OSCA) of Manila. The presence of MCI will be established following the criteria of Winblad et al. (2004) [42]. A person is diagnosed to have MCI if 1) the cognitive function is not normal nor demented; 2) there is subjective report of the individual and/or informant on declining cognitive function compared to five years ago [5] on objective cognitive tasks; and 3) the performance of basic ADLs is preserved with only minor problems doing complex instrumental ADLs [42]. Not normal cognitive function or presence of mild impairment will be based on Montreal Cognitive Assessment (MoCA) score of < 26 [43]. Not demented will be based on not having a medical diagnosis of dementia or Alzheimer’s disease [5, 44]. Normal function in ADL and no or minimal impairment in instrumental ADL will be based on Katz ADL Scale [45] and Lawton Instrumental Activities of Daily Living (IADL) Scale [4]. A trained neurologist-psychiatrist will examine the participants to provide the final diagnosis of MCI and give them the clearance to participate in the exercise. Only ambulatory older adults, with or without an assistive device, will be included since the interventions are community-based.

Older adults will be excluded if they have a diagnosis of dementia or Alzheimer’s disease; had history of major medical conditions such as cerebrovascular disease, cardiopulmonary condition, serious musculoskeletal disease, cancer, major psychiatric condition; have severe visual and/or hearing impairment; or are illiterate that will hinder them in participating fully and safely in the exercise and/or cognitive training programs. Furthermore, those who have been taking medications such as sedatives, antidepressants, diuretics, anti-epilepsy that might affect their cognitive function will not be included. Deliberately providing false information is a reason for termination of participation. Elevation of blood pressure beyond the expected increase due to exercise or the presence of fever and body pains for several days due to other causes may be reasons for withdrawal to participate. To enhance adherence to the intervention, participants will be receiving a travel allowance and light refreshments every time they attend sessions.

### Study design and procedures

This proposed study is a randomized controlled trial using a 4-group design including three intervention groups (physical training alone or cognitive training alone, or combined physical and cognitive training) and one waitlist control group. The study will comply with the principles of the Declaration of Helsinki 2013 and Good Clinical Practice Guidelines. Ethics approval was secured from the Human Subjects Ethics Sub-committee of the Hong Kong Polytechnic University-Department of Rehabilitation Sciences (HSEARS20170402001) and the Ethics Review Committee of the University of Santo Tomas-College of Rehabilitation Sciences (FI-2017-002). The SPIRIT guidelines were followed in the design of this study protocol.

Trained study personnel will interview the participants using questionnaires to obtain their demographic information and medical history, physical activity level and history of falls, and will conduct cognitive and physical performance tests to determine their eligibility to participate in this study. Only those who signed the informed consent will be included in this study.

Random allocation will be carried out by a third party not involved in the study by random draw to blindly allocate each participant to one of the four groups. To minimize experimental contamination through social interaction and communication among the participants, the interventions will be delivered at different sites.

**Fig. 1:**
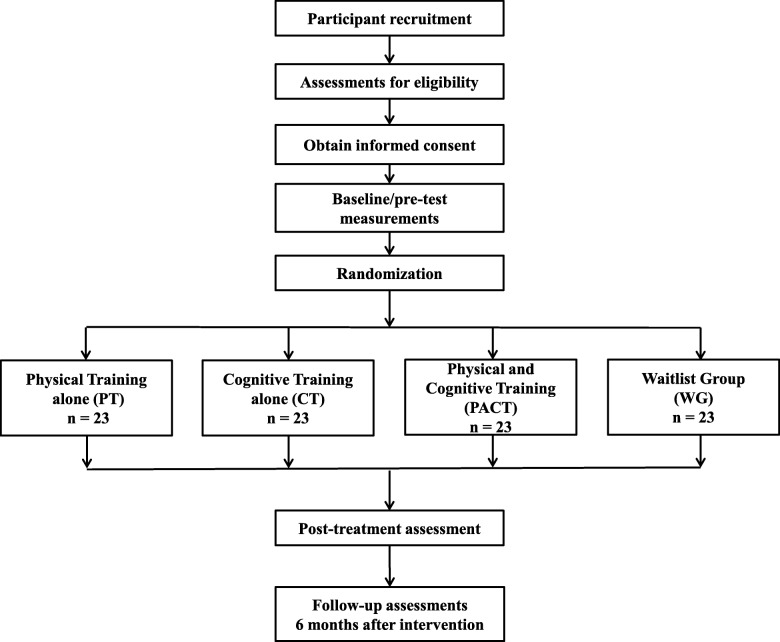
Flow diagram of the study

Measurement points include baseline/pre-intervention, post-intervention, and six months follow-up assessments and will be conducted by trained study personnel blinded to the randomization assignment. Physiotherapists and occupational therapists who will be administering the interventions will not be involved in the outcome assessment. They will also have workshops and discussions to standardize the administration of the different training programs. After the intervention period, aside from a calendar-diary, monthly phone calls will also be done to follow up the participants about their fall status, physical activity, and exercise behavior. Figure 1 illustrates the study flow. Appendix shows the timeline of the study.

### Sample size computation

The sample size and power calculations will be based on fall rate which is the primary outcome of the study. Using statistical software Gpower 3.1.9.2, a priori power analysis was conducted using the computed effect size (Cohen’s d = .39) from Trombetti et al. (2011) [46]. Assuming 80% power with 5% Type 1 error and four groups, the calculated total sample size is 80. Considering a 16% dropout rate (Trombetti et al., 2011) [46], the total number of participants is inflated to 93. There should be at least 23 participants per group.

### Interventions

#### Physical training alone (PT)

The participants in the PT group will be performing a series of physical exercises as a group supervised by trained physiotherapists. To ensure proper guidance and safety in the performance of the exercises, there will be one physiotherapist for every five participants.

The exercise program will start with 5–10 min of warm-up including calisthenics and general flexibility exercises, followed by 60–90 min of multicomponent exercise programme including endurance, strength and balance exercises (with a focus on balance training), and will end with 5–10 min of cool down including calisthenics and general flexibility exercises. This will be done three times per week over 12 weeks to achieve the recommended minimum accumulated 40 h of exercise [13]. The exercises will be progressed individually, increasing the repetitions or sets first before the resistance, to maintain a moderate level of exercise intensity which is 5 to 6 on a scale of 0 to10 for the level of physical exertion [47]. Rest periods will be provided whenever necessary. Table 1 shows the types of exercises included in this program.

**Table 1:** Physical training program

Periods	Exercises	Progression
Warm-up (5–10 min)	Calisthenics and general flexibility	
Multicomponent exercise programme (60–90 min)	Walking over 10 m 1. Walking forward-backward, sideways 2. Turning figure of 8 walking 3. Tandem walking	10–12 rounds; 1–3 sets Increase step length and speed Over obstacles
	Sit-to-stand	8–12 repetitions; 1–3 sets Lower chair height
	Heel and toe raises	8–12 repetitions; 1–3 sets Hold or raise for longer
	Stepping in different directions	8–12 repetitions; 1–3 sets Longer or faster steps Step over obstacle
	Step-ups 1. Forward 2. 2. Lateral	8–12 repetitions; 1–3 sets Increase step height
	Graded reaching in standing 1. Table top - side to side, forward and diagonal reaching 2. Tapping markers on the wall 3. 3. Reaching down to chair, stool or floor	8–12 repetitions; 1–3 sets Narrower foot placement Increase distance to reach Standing on a softer surface (rubber mat) Stepping while reaching
Cool-down (5–10 min)	Calisthenics and general flexibility	

The exercises were based on the recommendations of Sherrington and Tiedemann (2015) [48] and were selected because of the minimal use of sophisticated equipment which is important in an exercise regimen designed for a community setting. The participants will be given brochures containing simplified exercise instructions with illustrations and printed using a large font size to help them remember the exercise routines and encourage them to perform the exercises at home.

#### Cognitive training alone (CT)

The participants in the CT group will be involved in a set of paper-based cognitive exercises as a group supervised by trained occupational therapists. To provide close monitoring and immediate feedback during the training, there will be one occupational therapist for every five participants.

The intervention will start with 5–10 min of warm up to give the instructions, followed by 60–90 min of cognitive training designed to train specific cognitive functions such as orientation, memory, attention and executive function, with emphasis on executive function which is associated with increased risk for falls in older adults [25], and will end with 5–10 min of cool down for feedback and processing of responses. This will be done once a week for 12 weeks which is the optimal duration of cognitive training [30]. Even though the cognitive training is delivered in a group, the difficulty level and progression will be individualized. Rest periods will be provided whenever necessary.

Table 2 shows the contents of the cognitive training. The contents of training for orientation and attention were based from Brum et al. (2009) [49], and Vojtkofsky and Feldman (2015) [50]. For memory, training will include rehearsal, association, visual imagery and concentration. The use of multiple strategies in memory training is chosen as larger improvement is expected with this approach compared to single strategy approaches [50, 51]. For executive function, training will consist of extensive repeated practice of practical tasks to simulate usual daily activities instead of relying on strategies to strengthen cognitive processes. This is founded on the process-based approach which has been proven to be highly effective in improving executive function [27].

**Table 2:** Cognitive Training program

Periods	Components	Content
Warm-up (5–10 min)	Giving of instructions	
Cognitive training (60–90 min)	Orientation training	Orientation to person, place, and time with or without external cues like newspaper, calendar a. person – give full name, relatives, neighbors, age, occupation; name public officials b. place – provide address, location of islands/cities/provinces, favorite place/destination c. time – determine current time, day, month and year; schedule of TV shows; special occasions; weather, season
	Memory training	*Rehearsal* • verbally repeating a series of numbers or letters *Association* • face-name recognition – associating person’s name with facial or behavioral characteristics *Visual imagery* • having mental representation of a set of animals, fruits, or common objects and combinations *Concentration* • play card game to turn over 3–6 pairs of matching cards
	Attention training	*Auditory attention* a. Clapping, tapping or stamping upon hearing specific words b. Coloring a picture or folding a paper following dictation c. Identifying the title or artist of a song being played *Visual attention* a. Counting the number of animals seen in a picture b. Encircling specified words in a paragraph/ word hunt c. Completing a trace maze
	Executive function training	From a mixed set of objects on a table, a. Group the items into three and set aside objects which do not belong to any group. b. Arrange items following a model (e.g. table setting, smallest to largest, alternating pattern) Copying a drawing (pyramid, cylinder, house); dot copy Computation of allowance, expenses, change
Cool-down (5–10 min)	Feedback and processing of responses	

The paper-and-pencil format of cognitive training is selected in consideration of the low economic situation of the community. The participants will be given educational brochures with large fonts and illustrations to inform them about the importance of maintaining or improving their cognitive abilities citing specific ways to be mentally active in old age.

#### Physical and cognitive training (PACT)

The participants in the PACT group will be performing activities that integrate cognitive training in physical exercise routines as a group supervised by trained personnel either physiotherapists or occupational therapists. To ensure proper guidance, safety, close monitoring and immediate feedback about their performance of the activities, every five participants will be supervised by one therapist.

The PACT programme will start with 5–10 min of warm-up including calisthenics and general flexibility exercises, followed by 60–90 min of exercises similar in design, intensity and progression with the PT group but with cognitive components incorporated in each type of exercise, and will end with 5–10 min of cool down including calisthenics and general flexibility exercises and a post-session discussion to recall the activities performed in the just-concluded session. This will also be done three times per week over 12 weeks. Rest periods will be provided whenever necessary. Table 3 presents the design of the PACT program. The participants will likewise be given the same brochures given to the PT and CT groups for additional information and reference.

**Table 3:** Physical and cognitive training program

Periods	Exercises	Progression
Warm-up (5–10 min)	Calisthenics and general flexibility	
Multicomponent exercise programme (60–90 min)	Walking with executive function training • From a mixed set of objects (30 pieces) on a table at the start of the line, bring one object at a time walking over 10 m towards another table to sort the objects properly in 3–4 separate groups. Walking is done forward, backward, sideward, in figure of 8, or tandem.	10–12 rounds; 1–3 sets Increase step length and speed Walking over obstacles
	Sit-to-stand with orientation training • Stand every time to answer questions about orientation to person, place and time	8–12 repetitions; 1–3 sets Lower chair height
	Heel and toe raises with attention training • Follow visual cues to do heel or toe raises	8–12 repetitions; 1–3 sets Hold or raise for longer
	Stepping in different directions with memory training • Stepping on a set of specified number and sequence of markers on the floor	8–12 repetitions; 1–3 sets Longer or faster steps
	Step-ups with attention training • Follow verbal instructions on which foot to use to step-up	8–12 repetitions; 1–3 sets Increase step height
	Graded reaching in standing with executive function training a. Table setting activity (arrange plates, utensils, glasses based on picture model) b. Arranging objects from smallest to largest, or in alternating pattern c. Copying a drawing (pyramid, cylinder, house); dot copy d. Computation of allowance, expenses, change	8–12 repetitions; 1–3 sets Narrower foot placement Increase distance to reach Standing on a softer surface (rubber mat) Stepping while reaching
Cool-down (5–10 min)	Calisthenics and general flexibility With memory training (summarize what activities were accomplished after the session in correct sequence and details)	

#### Waitlist group (WG)

The participants in the WG will serve as the control group on waitlist. They will be instructed to go on with their usual daily routine and will receive the intervention, combined physical and cognitive training, at a later date.

### Outcomes measures

The following primary and secondary outcome measures will be performed by trained assessors blinded to the group allocation. Measurements will be taken at baseline, at the conclusion of the intervention period, and 6 months post-intervention. Additional information regarding the exercise behavior and the physical and social activities participated in by the eligible participants in the last 6 months will be obtained during the 6 months post-intervention follow-up assessments [52].

### Primary outcome measure

#### Falls rate

Each participant will be given a calendar diary to mark any incidence of fall on a weekly basis during the assessment period. A relative or caretaker of the older person will be asked to validate the information. The confirmation by another person is done because self-reporting techniques, particularly for individuals with cognitive impairment, may not be accurate due to recall bias [53]. Monthly phone calls will be done to update the falls history of each participant.

### Secondary outcome measures

The secondary measures will be assessments that evaluate overall fall risk, cognitive, physical and psychological factors related to falls.

#### Overall fall risk

The overall fall risk will be determined using the short-form Physiological Profile Assessment (PPA) and the Falls Risks for Older People in the Community (FROP-Com) Screen.

The PPA short form is a valid, reliable and objective series of tests used to evaluate the physiologic risks of falls and to classify fallers from non-fallers [54]. It is composed of five subtests on 1) visual contrast sensitivity, 2) proprioception, 3) hand reaction time, 4) knee extensor muscle strength, and 5) postural sway [55]. Data from these tests will be encoded in a web-based software program such that the performance of one participant is compared to a normative database to determine whether the individual has a low or high risk of falls [55].

The FROP-Com Screen is a brief screening tool used to determine older persons who are prone to fall [56]. It is composed of three items on 1) the number of incurred falls within the past 12 months, 2) the level of dependence in doing instrumental ADLs, and 3) balance in walking and turning of older adults [56]. A score of 0–3 means the older adult has a low risk for falls, while a score from 4 to 9 denotes a high risk for falls [56].

#### Cognitive function

Montreal Cognitive Assessment (MoCA) is a brief 10-min, 30-item, one-page cognitive screening, diagnostic and tracking tool with high sensitivity and specificity for detecting MCI [43, 57]. It is widely used internationally and has been translated into many languages. It assesses several cognitive domains such as memory, executive function, attention, language, abstraction, naming, delayed recalls, and orientation.

#### Memory

The Memory Index Score of the Montreal Cognitive Assessment (MoCA-MIS) consists of 15 items on memory and validated to help determine in predicting conversion to Alzheimer’s disease or dementia from MCI over 18 months [58]. It is calculated by getting the sum of the number of words correctly remembered by the participant in free delayed recall, category cued recall, and multiple choice-cued recall multiplied by 3, 2 and 1, respectively, to obtain a score which ranges from 0 to 15 [58].

#### Executive function

Executive Function Performance Test (EFPT) is a valid and reliable instrument that uses structured cueing and scoring system to examine executive function in doing basic real-world tasks like hand-washing, oatmeal preparation, telephone use, taking medication, and paying bills [59]. To consider the local context, only the use of the telephone and taking of medication will be performed in this study.

#### Balance

The Timed Up and Go Test (TUGT) will be used to examine the dynamic balance of the participants. It is a simple and reliable [60] test that measures the time in seconds it takes a person to stand up from a chair, walk a distance of three meters, turn around, walk back towards the chair and sit down again. It is a sensitive and specific tool for identifying older adults who are at risk of falls [61]. Those who take longer than 14 s to complete this test have a high risk of falls [61].

#### Gait speed

Gait speed will be measured using the 10-Meter Walk Test (10MWT). This is a valid and reliable gait speed assessment in older adults [62]. It is the quotient of distance covered in meters, and the duration in seconds it takes the participant to walk that distance. The participants will perform the test at their preferred speed and fastest speed possible [62, 63].

#### Muscle strength

The strength of the lower limb muscles will be assessed using the 30s-Chair-Stand Test (CST). This is done by asking the participants to perform sit-to-stand on a standardized chair as many as they can within 30 s. The CST has excellent validity and reliability in community-dwelling older persons [64].

#### Fear of falling

The Falls Efficacy Scale – International (FES-I) will be used to assess the fear of falling of the participants. The tool is composed of 16 items. Each item is scored based on a four-point scale (1 = not at all concerned; 2 = somewhat concerned; 3 moderately concerned; and 4 = very concerned). The score ranges from 16 to 64. A high score means more fear of falling. FES-I has been shown to have excellent psychometric properties including construct validity, internal consistency, and test-retest reliability and discriminatory power when used in older persons [65–68].

#### Quality of life

Perceived Well-Being (PWB) Scale is a brief and easy to use tool that measures the quality of life specifically in the domains of psychological and physical well-being. It has high internal consistency and validity and is significantly correlated with variables that affect an individual’s well-being [69]. The score ranges from 16 to 116. A high score means greater perceived well-being.

#### Health status

EuroQoL-5 dimensions-5 levels (EQ-5D-5 L) is a valid and standardized tool developed to provide a simple, generic measure of health status for clinical and economic analysis [70]. The five dimensions are mobility, self-care, usual activities, pain/discomfort, and anxiety/depression. Each dimension has five levels: no problems, slight problems, moderate problems, severe problems, and extreme problems [71]. The tool has shown evidence of responsiveness to change [72]. It has been translated into over 120 languages and the validated Tagalog version of EQ-5D-5 L will be used with permission in this study.

### Statistical analysis

Descriptive statistics will be used to summarize demographic data using mean standard deviations, and percentages. Baseline values across the groups will be compared using one-way ANOVA for interval/ratio data and Kruskal-Wallis test for nominal/ordinal data. The multivariate repeated measure ANOVA will be used to determine the pre- and post-intervention effects and differences among group data with a normal distribution; otherwise the Friedman test will be utilized. The incidence rate ratio, computed as the number of falls divided by the duration of falls monitoring for every participant, will be used to compare intergroup fall rate. An intention-to-treat analysis will be used for missing data due to dropouts. The *p*-value of < 0.05 is considered significant. Data analysis will be performed using SPSS version 23 for Windows.

### Data monitoring

Collected data using a paper-based assessment kit will be encoded in MS Excel by one study personnel blinded to the group allocations. This will be double-checked by another research assistant. The privacy and confidentiality of the participants will be kept by making no personal information available to the public. Signed consent forms and filled data collection sheets will be stored in a locked cabinet. The encoded data will be kept in a password-protected computer folder. Data will be stored for five years and will then be deleted. In the event of any publication involving this study, the participants’ identities will remain confidential. Aside from the researchers, the study monitors from the ethics committee will be granted monthly direct access to the records for purposes of procedure and data verification and interim analyses. Any modifications in the study procedure will be reported to the Human Subjects Ethics Sub-committee of The Hong Kong Polytechnic University-Department of Rehabilitation Sciences and the Ethics Review Committee of the University of Santo Tomas-College of Rehabilitation Sciences, and to ClinicalTrials.gov. To disseminate the findings of this research, the results and implications will be reported to the participants and presented formally in professional conferences, and published in a reputable journal.

### Adverse events

Participants might experience fatigue while doing exercise, and therefore adequate rest periods and breaks will be provided as needed. Vital signs will be constantly monitored before, during and after the interventions every session. Light refreshments will be provided to prevent dehydration. In some activities that will challenge their balance, trained therapists will be close by to provide support to prevent them from falling. The calendar-diary will also be used to monitor changes in health status and record adverse events every session during the intervention period and then every month after the intervention phase. In the case of any untoward incident like dizziness, injuries, or falls during the conduct of the study, appropriate first aid, medical attention or referral to a clinic or hospital, will be immediately done to the participant as soon as reported.

## Discussion

The objective of this proposed study is to determine the effectiveness of combined physical and cognitive training on preventing falls and reducing the risk of falls among community-dwelling older adults with MCI. To our knowledge, this is the first trial that will investigate the direct effect of combined physical exercise and cognitive training on falls in this target population [73].

A fall could result in fatal or non-fatal physical injuries [53, 74], psychosocial problems [75], and also brings about economic burden to the family and community [76–78]. Older people with MCI are at a heightened risk for falls [1]. It is essential, therefore, to scientifically determine which treatment programs are most effective in this population [79].

A previous study reported that all aspects of balance control deteriorate with increasing severity of cognitive impairment and that executive function plays an important role in balance control [80]. Early intervention, therefore, is vital for the care of older people with MCI to prevent or retard its progression to dementia and to prevent the occurrence of falls [2, 26]. This study will contribute knowledge on the impact of proactive measures in older persons with MCI.

We are anticipating that since the training programs will run for 12 weeks, we might have difficulty recruiting participants and also sustaining their attendance throughout the intervention period. To address this, the researchers will be working closely together with the local OSCA coordinators in inviting participants and following them up. Travel allowance and refreshments will also be provided to the participants every time they attend a session to encourage them to be diligent in their involvement. Participants may also drop-out due to various reasons (busy schedule, relocation, low motivation, diseases) which is why the sample size was inflated by 16% [46].

In summary, this trial will provide insight into the effect of integrating cognitive training into physical exercise in preventing falls and reducing physical, cognitive and psychologic risks of falls in older persons with MCI living in the community. Healthcare professionals and practitioners in the geriatric field will be provided with validated community-based interventions whose effect on falls and fall-related risk factors is scientifically evaluated.

## Appendix

**Table 4:** Timeline of the study

Research Highlights	Actual/Expected dates of implementation
Participant recruitment	June–September 2017
Baseline assessment	August–September 2017
Randomization	September 2017
Post-treatment assessment	December 2017–January 2018
9mons post baseline assessment	June–July 2018
Study completion	August 2018

## Abbreviations

10MWT: 10-Meter Walk Test
CST: 30s-Chair-Stand Test
CT: Cognitive Training
EF: Executive Function
EFPT: Executive Function Performance Test
FES-I: Falls Efficacy Scale – International
FROP-Com: Falls Risk for Older Persons in the Community
MCI: Mild cognitive impairment
MoCA: Montreal Cognitive Assessment
MoCA-MIS: Memory Index Score of the Montreal Cognitive Assessment
PACT: Physical And Cognitive Training
PPA: Physiologic Profile Assessment
PT: Physical Training
PWB: Perceived Well-Being
TUGT: Time Up and Go Test
WG: Waitlist Group
